# Solid Pseudopapillary Neoplasm of Pancreas in a 26‐Year‐Old Female: A Case Report and Comprehensive Review of Literature

**DOI:** 10.1002/ccr3.71218

**Published:** 2025-10-10

**Authors:** Salina Uprety, Suraj KC, Shivam Pandey, Sushma Jaishi, Ramesh Sapkota, Sahil Niraula, Bhawani Khanal, Rakesh Kumar Gupta

**Affiliations:** ^1^ Department of General Surgery B.P. Koirala Institute of Health Sciences Dharan Nepal

**Keywords:** β‐catenin, distal pancreatectomy, multivisceral resection, solid pseudopapillary neoplasm

## Abstract

Solid pseudopapillary neoplasm (SPN) is a rare, low‐grade malignant tumor of the pancreas that predominantly affects young females. Although SPN is usually slow‐growing and benign, it can exhibit local invasion or metastasis, emphasizing the need for prompt diagnosis and treatment. SPNs typically manifest as painless abdominal masses or are incidentally discovered on imaging. While most SPNs follow a benign course, they can invade nearby structures and, in rare instances, metastasize to the liver, omentum, or peritoneum. We report a rare case of a 26‐year‐old female who presented with a painless abdominal mass that had been growing for 8 years. Imaging revealed a heterogeneous pancreatic mass with signs of local invasion. The patient underwent distal pancreatectomy with en bloc splenectomy and segmental colectomy. This case highlights the importance of considering SPN in the differential diagnosis of abdominal masses in young females and emphasizes the role of surgical resection in achieving favorable outcomes, even in cases with extensive local spread.

**Summary**

- Solid pseudopapillary neoplasm (SPN) of the pancreas presents a diagnostic challenge due to its rare occurrence, nonspecific symptoms, and variable clinical course.
- Though the tumor primarily affects young women, it can remain asymptomatic and hence difficult to diagnose.
- Despite its typically slow growth, SPNs can exhibit local invasiveness, underscoring the need for evaluation.
- Imaging techniques like contrast‐enhanced CT and MRI are valuable for preoperative assessment and operability, but a definitive diagnosis relies on histopathological and immunohistochemical analysis, particularly demonstrating nuclear β‐catenin positivity.
- Complete surgical removal is the mainstay of treatment and offers excellent prognosis, even for large locally invasive tumors.
- This case adds to the existing literature on SPNs and underscores the importance of a multidisciplinary approach, high index of suspicion, timely surgery by initial diagnostic laparoscopy to rule out metastases, definitive surgical resection, and long‐term monitoring to achieve optimal outcomes for patients.

## 1 Introduction

Solid pseudopapillary neoplasms (SPNs) are the least common subtype of pancreatic cystic neoplasms, accounting for 1%–3% of all pancreatic tumors and 10%–15% of pancreatic cystic neoplasms. Initially described in 1959, they were classified as solid pseudopapillary tumors (SPTs) by the WHO in 1996 and are now termed SPNs [1]. The European expert consensus focuses on and concludes that intraductal papillary mucinous neoplasm, mucinous cystic neoplasm, serous cystic neoplasm, and SPTs fall under 90% of all cystic tumors of the pancreas. The characteristics of SPTs are unique from those of pancreatic cancer in terms of their age of occurrence and gender predisposition. All SPTs should be resected as they have unequivocal malignant potential; also, in view of their favorable prognosis, surgery remains the mainstay of treatment [2]. We report a 26‐year‐old female who presented with an enlarging, painless abdominal mass over 8 years. Radiological imaging suggested a heterogeneous pancreatic mass with local invasion. She underwent distal pancreatectomy with en bloc splenectomy and segmental colectomy. Histopathological examination confirmed SPN, with immunohistochemical positivity for β‐catenin and androgen receptor. This case underscores the importance of considering SPN in the differential diagnosis of abdominal masses in young females and highlights the role of surgical resection in achieving favorable outcomes, even in cases with extensive local spread.

The work has been reported in line with the SCARE 2023 criteria [3].

## 2 Case History and Examination

A 26‐year‐old nulliparous female, with no significant past medical or surgical history, presented to our surgical outpatient department with complaints of a gradually enlarging mass in the left flank over the past 8 years. The mass was initially small, asymptomatic, and slowly increased in size. There was no associated pain, fever, anorexia, weight loss, nausea, vomiting, or change in bowel or bladder habits. The patient had no other symptoms for 8 years besides swelling, which led her to seek medical care late. The patient denied any history of trauma, hormonal therapy, smoking, or alcohol consumption. Menstrual history was unremarkable, and there was no family history of malignancy. On physical examination, the abdomen was soft and non‐tender with a palpable, firm, non‐pulsatile, mobile mass measuring approximately 10 × 5 cm in the left flank, extending toward the supraumbilical region. The mass exhibited a positive cough impulse, raising a preliminary suspicion of a mesenteric or retroperitoneal lesion.

## 3 Differential Diagnosis, Investigations and Treatment

In regard to the provided history, we thought it to be swelling either arising from the stomach, pancreas, or kidney, so we performed investigations accordingly. Baseline laboratory investigations, including complete blood count, liver and renal function tests, serum amylase, and lipase, were within normal ranges. Serum tumor markers—carcinoembryonic antigen (CEA: 3 ng/mL) and carbohydrate antigen 19‐9 (CA 19‐9: 15 ng/mL) were not elevated. Ultrasound of the abdomen revealed a large, well‐circumscribed heteroechoic lesion in the left hypochondrium extending into the epigastrium. To further delineate the anatomy, contrast‐enhanced computed tomography (CECT) of the abdomen and pelvis was performed.

**FIGURE 1:**
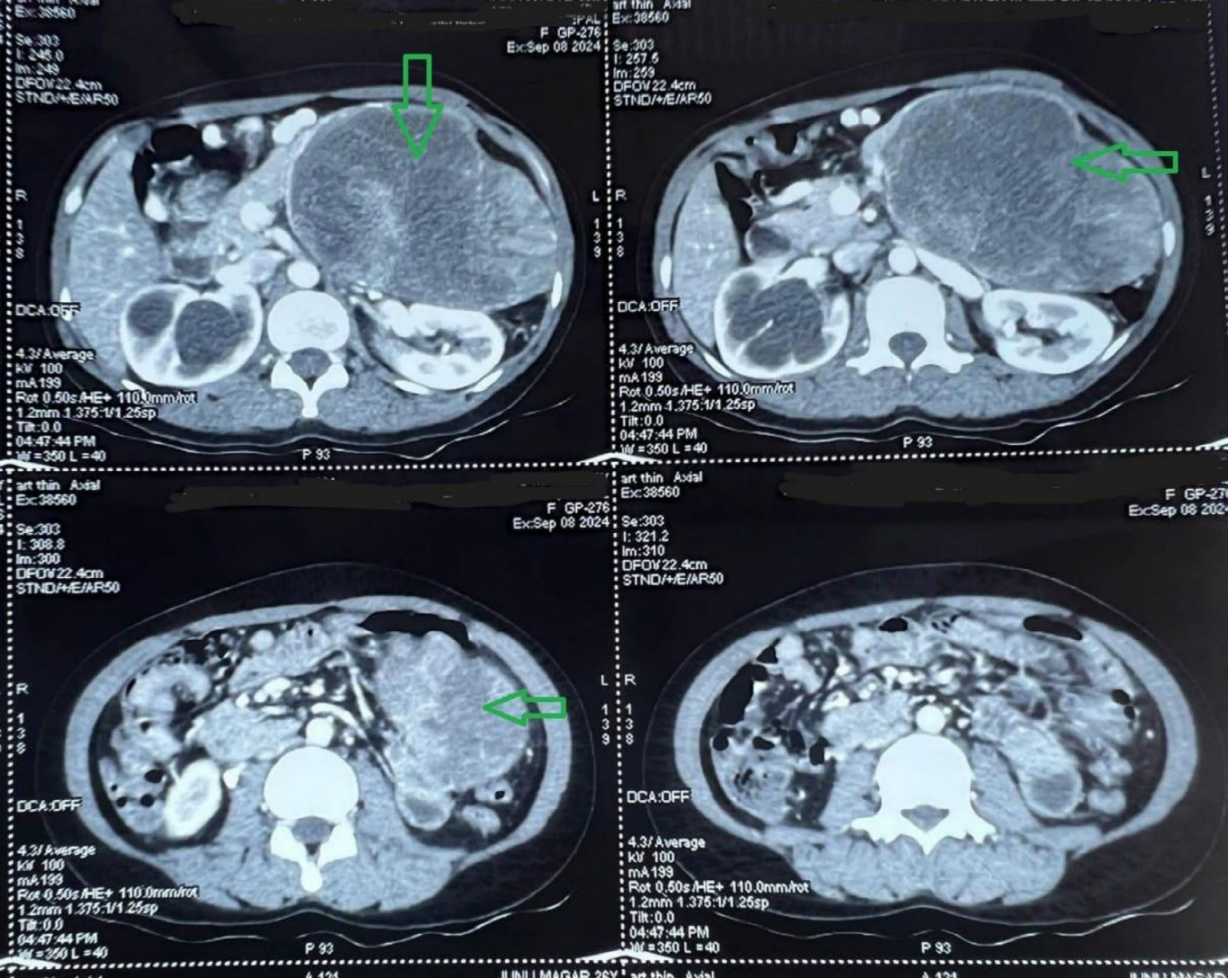
Contrast‐enhanced CT scan of the abdomen showing a large, well‐encapsulated heterogenous mass arising from the body and tail of the pancreas, with solid and cystic components, internal hemorrhage, and invasion into the spleen and splenic flexure of the colon. The right‐sided pelvicalyceal system is also dilated.

CECT revealed a multiloculated, encapsulated mass measuring approximately 12.06 × 11.84 × 9.95 cm arising from the body and tail of the pancreas. The lesion demonstrated both solid and cystic components with internal hemorrhagic areas. There was invasion of the adjacent spleen and compression of the splenic vein, resulting in collateral venous formation. The mass appeared adherent to the splenic flexure of the colon and abutting the upper pole of the left kidney. No evidence of distant metastasis was noted (Figure 1). Based on clinical and radiological findings, a provisional diagnosis of a SPN of the pancreas was made. The patient was planned for elective surgery. Intraoperatively, the tumor was found to be arising from the body and tail of the pancreas, with invasion into the spleen and splenic flexure of the colon. The decision for a multivisceral en bloc resection was made. She underwent distal pancreatectomy with en bloc splenectomy and segmental resection of the splenic flexure. Bowel continuity was restored using a colo‐colic end‐to‐end anastomosis.

## 4 Outcomes and Follow Up

The postoperative course was uneventful. The patient was discharged on the sixth postoperative day with advice for regular follow‐up. She remains asymptomatic and is currently under surveillance by the surgical oncology team.

**FIGURE 2:**
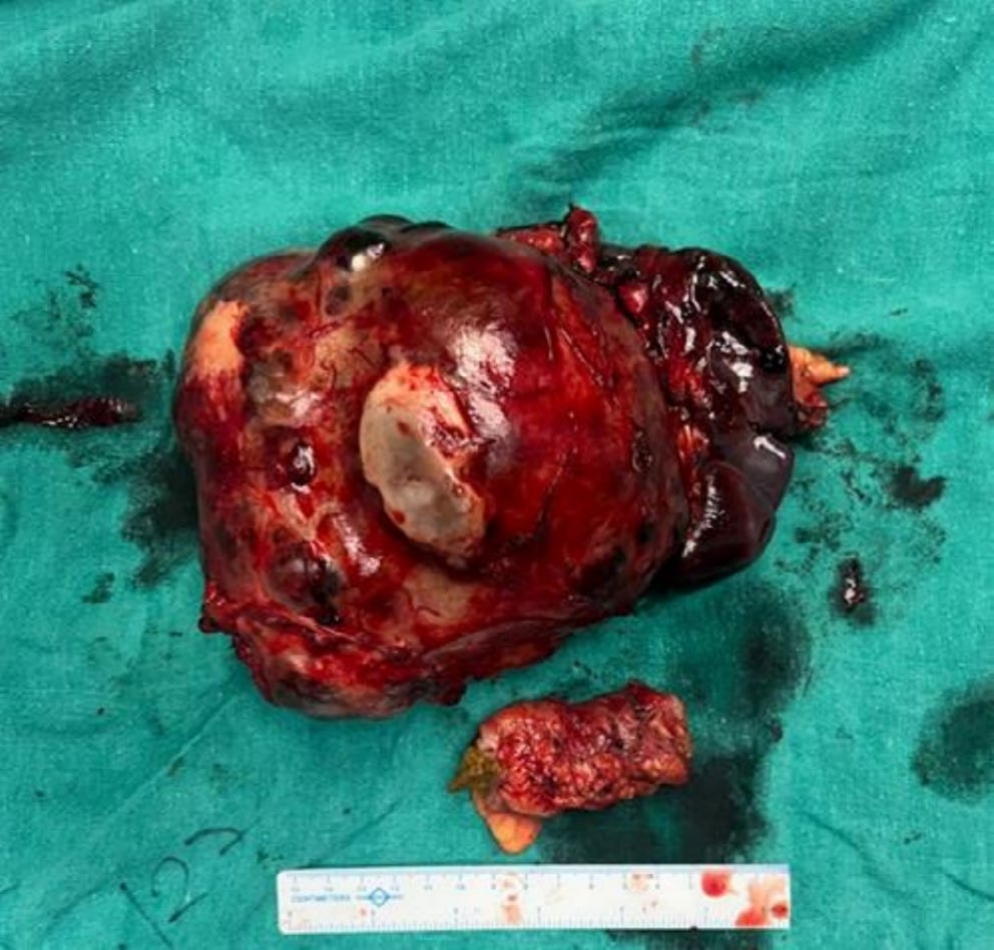
Resected specimen showing a large encapsulated pancreatic mass with heterogeneous cut surface and hemorrhagic areas. Adjacent spleen and segment of colon are also seen as part of the en bloc resection.

**FIGURE 3:**
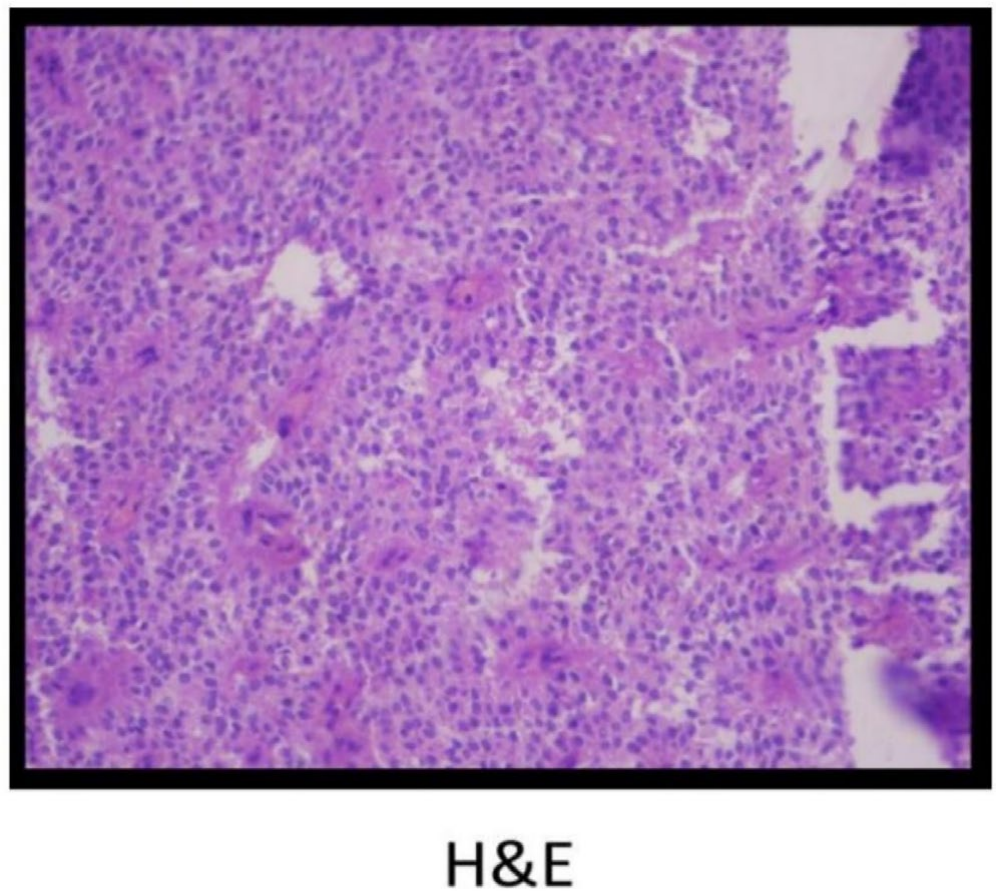
Hematoxylin and eosin (H&E) staining at 400× magnification showing pseudopapillary architecture with uniform polygonal tumor cells, fibrovascular cores, and areas of hemorrhage.

**FIGURE 4:**
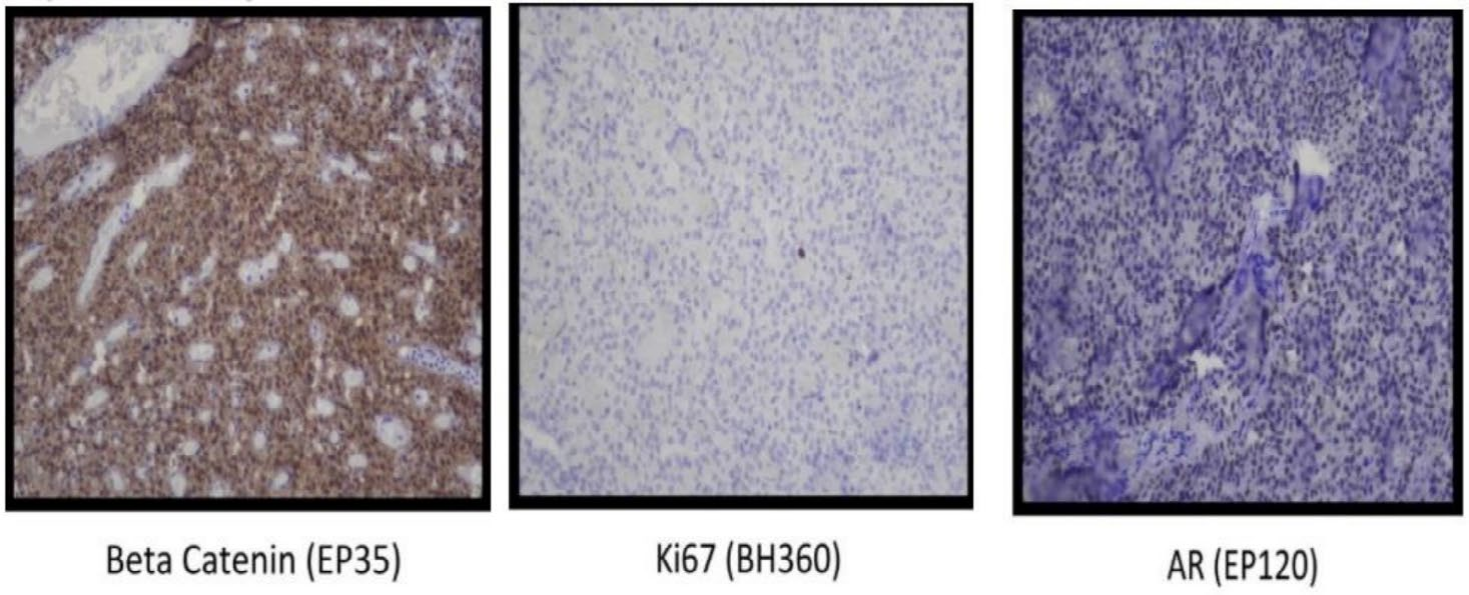
Immunohistochemical staining of the tumor: Strong nuclear and cytoplasmic positivity for β‐catenin (EP35), low Ki‐67 proliferation index (BH360), and positive androgen receptor (AR, EP120) expression. These findings support the diagnosis of SPN.

Gross pathological examination of the resected specimen showed a 16 × 16 × 8 cm encapsulated tumor with heterogeneous appearance, containing solid and cystic areas with focal hemorrhage (Figure 2). Histopathology revealed a neoplasm composed of monomorphic polygonal cells arranged in solid sheets and pseudopapillary structures. Hyalinized stroma and focal necrosis were present (Figure 3). Immunohistochemical staining showed strong nuclear and cytoplasmic positivity for β‐catenin, as well as positive staining for SOX11 and androgen receptor (AR). The tumor cells were negative for cytokeratin, synaptophysin, chromogranin A, and progesterone receptor (PR), confirming the diagnosis of a SPN of the pancreas (Figure 4).

## 5 Discussion

Pancreatic SPTs are a rare clinical entity, with low malignancy and an unclear pathogenesis. Hypotheses suggest their origins from pluripotent pancreatic cells or the female genital bud [2]. The fact that SPTs predominantly affect young women compelled several authors to study the role of hormonal receptors, but no evidence of estrogen receptors in the pathogenesis of the tumor has been found. The p53 gene and k‐ras also do not seem to play an important role in the pathogenesis of SPTs. It has been found that the pathogenesis is linked to β‐catenin mutations and Wnt signaling pathway dysregulation [4]. SPTs predominantly affect young women in their second or third decades of life, with a striking female‐to‐male ratio of 7:1, a mean age of 28.5 years, and tumor sizes averaging 8.6 cm [5]. Most cases are located in the body or tail of the pancreas (62.4%), followed by the head or neck (37.6%). Extra‐pancreatic cases are rare (1.1%) [2]. Clinical presentation is often non‐specific, with over half of patients experiencing abdominal pain or discomfort, while others present with a palpable mass, nausea, or weight loss. Interestingly, one‐third are asymptomatic at diagnosis [2, 5]. Unlike other pancreatic malignancies, jaundice, significant weight loss, and pancreatitis are uncommon [5]. On computer tomography (CT) imaging, SPENs characteristically are thick‐walled encapsulated structures with peripheral solid and central cystic components. An important differentiating feature from other pancreatic neoplasms is that SPENs enhance similarly to surrounding pancreatic parenchyma with contrast [6]. However, magnetic resonance imaging (MRI) is considered superior to CT in diagnosing SPENs. On MRI, SPENs appear heterogeneous with varying signal intensity on T1‐weighted imaging and heterogeneous with high intensity on T2‐weighted imaging [7]. Fine‐needle aspiration, performed percutaneously or with endoscopic ultrasound guidance, aids in the preoperative diagnosis [5]. Despite a low malignant potential, SPNs can exhibit vascular invasion (4.6%), lymph node involvement (1.6%), and distant metastases (7.7%), most commonly to the liver or spleen [4, 8]. Local invasion, recurrence, or limited metastases do not preclude surgical resection, and long‐term survival exceeding 10 years is possible [4]. Surgery is the cornerstone of SPN treatment, with procedures including distal pancreatectomy, pancreaticoduodenectomy, enucleation, and total pancreatectomy. Adjuvant chemotherapy or radiotherapy may benefit non‐resectable cases, improving prognosis [5]. Risk factors for recurrence include incomplete resection, large tumor size, younger age, tumor rupture, and male sex [2]. Grossly, these tumors display a solid, cystic, and hemorrhagic morphology. Histological analysis reveals uniform cells forming solid sheets, with pseudopapillae arising from cell detachment. Hemorrhage, foam cells, cholesterol granulomas, and entrapped pancreatic tissue are frequently observed [9]. Immunohistochemically, SPNs are positive for vimentin, α1‐antitrypsin, neuron‐specific enolase, CD10, and CD56 [4, 8]. SPNs have an excellent prognosis. Overall, five‐year survival is as high as 97% in patients undergoing surgical resection, even in the case of distant hepatic metastasis or local recurrence [10]. SPENs are radiosensitive and show favorable responses to systemic chemotherapy and hormonal therapy. Chemotherapeutic regimens include floxuridine, oxaliplatin, etoposide, and cisplatin [6, 11].

## 6 Conclusion

Solid pseudopapillary neoplasm of the pancreas presents a diagnostic and treatment challenge due to its rare occurrence, nonspecific symptoms, and variable clinical course. This tumor primarily affects young women and can remain asymptomatic for an extended period, complicating early detection. Despite its typically slow growth, SPNs can exhibit local invasiveness, underscoring the need for vigilance when assessing abdominal masses in young females. Imaging techniques like contrast‐enhanced CT and MRI are valuable for preoperative assessment, but a definitive diagnosis relies on histopathological and immunohistochemical analysis, particularly demonstrating nuclear β‐catenin positivity. Complete surgical removal is the mainstay of treatment and offers a favorable prognosis, even for large or locally invasive tumors. This case adds to the existing literature on SPNs and underscores the importance of a multidisciplinary approach, timely surgery, and long‐term monitoring to achieve optimal outcomes for patients.

## Author Contributions

**Salina Uprety:** data curation, formal analysis, project administration, supervision, writing – review and editing. **Suraj KC:** conceptualization, data curation, project administration, resources, visualization, writing – original draft, writing – review and editing. **Shivam Pandey:** methodology, resources, writing – original draft. **Sushma Jaishi:** conceptualization, investigation, resources, supervision. **Ramesh Sapkota:** conceptualization, methodology, resources, supervision. **Sahil Niraula:** methodology, resources, writing – original draft. **Bhawani Khanal:** data curation, formal analysis, investigation. **Rakesh Kumar Gupta:** conceptualization, data curation, formal analysis, investigation.

## Consent

Informed written consent was obtained from the patient for publication of this case report.

## Conflicts of Interest

The authors declare no conflicts of interest.

## Data Availability

The data that support the findings of this study are available from the corresponding author upon reasonable request.
